# 2412. Whole-body PET scans for infective endocarditis: differences in patient characteristics and outcomes

**DOI:** 10.1093/ofid/ofad500.2032

**Published:** 2023-11-27

**Authors:** Kaitlyn Simpson, Jason Park, Jamie Riddell

**Affiliations:** University of Michigan Medical School, Ann Arbor, Michigan; Michigan Medicine, Ann Arbor, Michigan; University of Michigan, Ann Arbor, MI

## Abstract

**Background:**

Whole-body 18F-FDG PET/CT (WBP) has the potential to impact management of infective endocarditis (IE) through identification of areas of occult primary or metastatic infection. This study compares the characteristics and outcomes in groups of patients with IE who did and did not have WBP as part of their evaluation to help guide how this imaging may be optimally utilized.

**Methods:**

We performed a retrospective cohort study of hospitalized patients discussed at the multidisciplinary endocarditis meeting at a tertiary care center in Michigan, USA between June 2018 and January 2022. Protocolized chart review was performed by three study team members.

**Results:**

There were 114 (26.7%) patients with suspected IE who had WBP and 313 (73.3%) who did not. The two groups were similar in age, gender, and race. Patients with ESRD on dialysis (p=0.01), cardiac and/or ascending aortic prosthetic material (p< 0.001) or endocardial devices (p=0.01), and those who had cardiac PET (P< 0.001) were more likely to have undergone WBP. Complications such as paravalvular abscess (p=0.004) and flail leaflet (p=0.01) were more common in the non-WBP group. There was no difference in surgical indication between groups, though the WBP group had a non-significant trend of not receiving cardiac surgery (p = 0.05). WBP was associated with a longer-course of antibiotics (6.36 vs 5.32; p< 0.001) and suppressive antibiotics (30.70% versus 13.42%; p< 0.001). Within the WBP group, 45.61% had a change in management after imaging with the most common changes being further infectious (20.18%) or non-infectious (9.65%) workup, source control procedure performed (10.52%), and antibiotic regimen change (9.64%). No other outcomes between groups were significantly different between groups including length of stay, 6-month readmission, 6-month infection relapse, 6-month mortality or in-hospital mortality.

**Figure:**
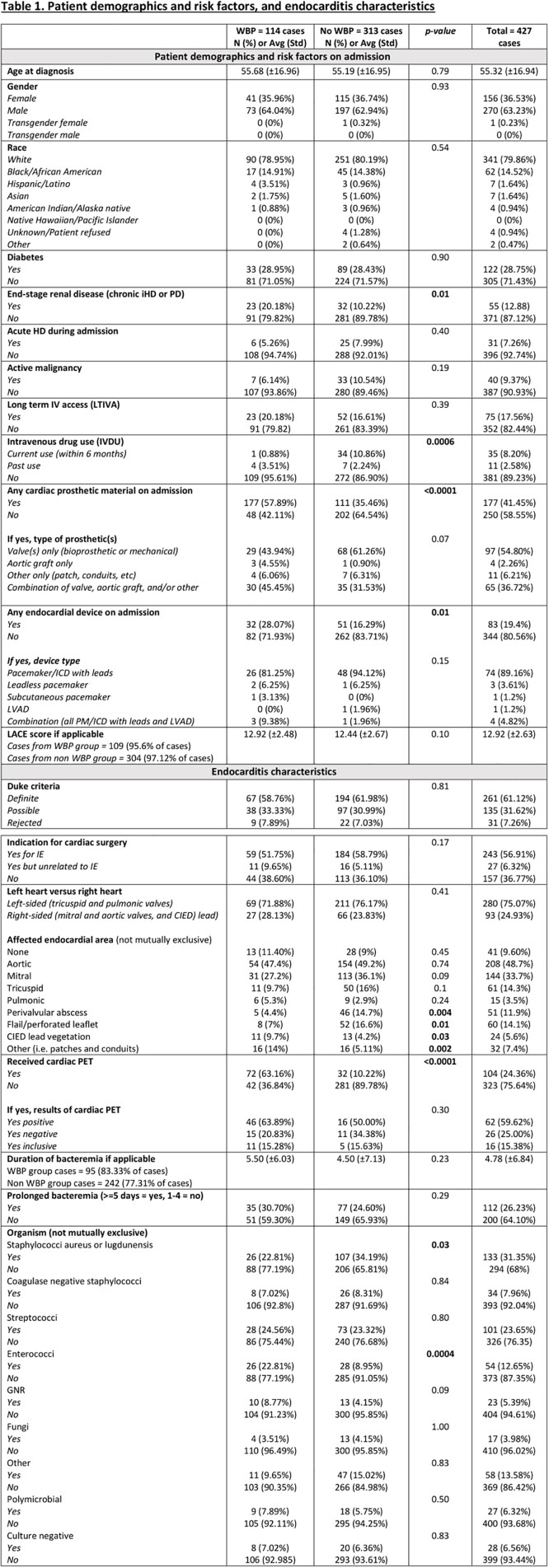

**Figure:**
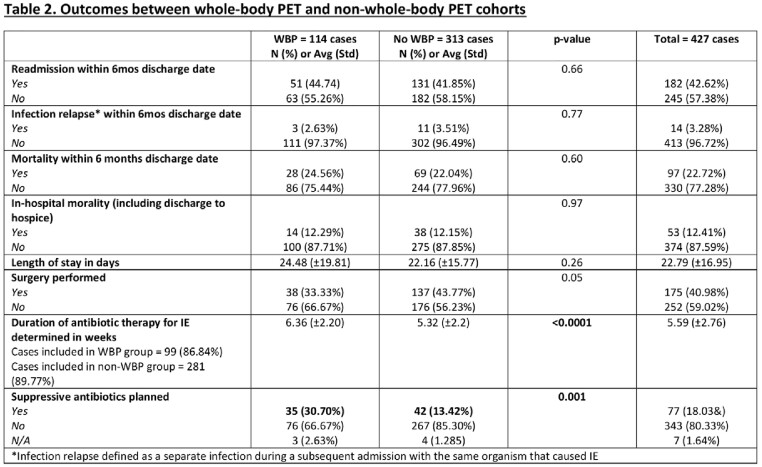

**Figure:**
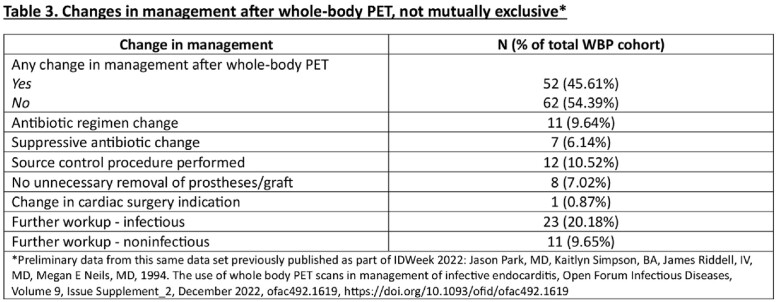

**Conclusion:**

Although a mortality benefit was not observed, WBP played an important role in the management of patients with IE with identification of areas of metastatic infection that required longer antimicrobial treatment or addition of suppressive antimicrobials. Further analyses are planned to understand which patients with IE may benefit most from WBP.

**Disclosures:**

**All Authors**: No reported disclosures

